# Implementation frameworks for end-to-end clinical AI: derivation of the SALIENT framework

**DOI:** 10.1093/jamia/ocad088

**Published:** 2023-05-19

**Authors:** Anton H van der Vegt, Ian A Scott, Krishna Dermawan, Rudolf J Schnetler, Vikrant R Kalke, Paul J Lane

**Affiliations:** Centre for Health Services Research, The University of Queensland, Brisbane, Australia; Department of Internal Medicine and Clinical Epidemiology, Princess Alexandra Hospital, Brisbane, Australia; Centre for Information Resilience, The University of Queensland, St Lucia, Australia; School of Information Technology and Electrical Engineering, The University of Queensland, St Lucia, Australia; Patient Safety and Quality, Clinical Excellence Queensland, Queensland Health, Brisbane, Australia; Safety Quality & Innovation, The Prince Charles Hospital, Queensland Health, Brisbane, Australia

**Keywords:** AI framework, AI implementation, machine learning, artificial intelligence, healthcare framework

## Abstract

**Objective:**

To derive a comprehensive implementation framework for clinical AI models within hospitals informed by existing AI frameworks and integrated with reporting standards for clinical AI research.

**Materials and Methods:**

(1) Derive a provisional implementation framework based on the taxonomy of Stead et al and integrated with current reporting standards for AI research: TRIPOD, DECIDE-AI, CONSORT-AI. (2) Undertake a scoping review of published clinical AI implementation frameworks and identify key themes and stages. (3) Perform a gap analysis and refine the framework by incorporating missing items.

**Results:**

The provisional AI implementation framework, called SALIENT, was mapped to 5 stages common to both the taxonomy and the reporting standards. A scoping review retrieved 20 studies and 247 themes, stages, and subelements were identified. A gap analysis identified 5 new cross-stage themes and 16 new tasks. The final framework comprised 5 stages, 7 elements, and 4 components, including the AI system, data pipeline, human-computer interface, and clinical workflow.

**Discussion:**

This pragmatic framework resolves gaps in existing stage- and theme-based clinical AI implementation guidance by comprehensively addressing the what (components), when (stages), and how (tasks) of AI implementation, as well as the who (organization) and why (policy domains). By integrating research reporting standards into SALIENT, the framework is grounded in rigorous evaluation methodologies. The framework requires validation as being applicable to real-world studies of deployed AI models.

**Conclusions:**

A novel end-to-end framework has been developed for implementing AI within hospital clinical practice that builds on previous AI implementation frameworks and research reporting standards.

## INTRODUCTION

Modern healthcare is underpinned by the translation of research findings into clinical practice. Regulatory practices in most countries aim to minimize the risks associated with introducing new technologies such as drugs and medical devices. Honest and accurate appraisal of new technologies is also encouraged by clinical researchers adhering to reporting standards.^1^^,^^2^ However, despite prolific growth in research into artificial intelligence (AI) based decision support technologies over recent years,^3^ particularly diagnostic and prognostic prediction models, translation into clinical practice has been slow^4^^,^^5^ and the numbers of AI-based systems of this type implemented into routine care remain very low.^6^^,^^7^ In a recent scoping review, just 45 of these AI systems had been implemented over 10 years,^8^ compared to over 15 000 published research papers on AI in healthcare in 2020 alone.^3^

The reasons for slow uptake are multiple, including lack of clinician trust in often-unexplainable and opaque “black-box” AI methods,^9–13^ consumer fears over data privacy,^14^^,^^15^ health inequity concerns about potential underlying data biases^13^^,^^16^ and underdeveloped or absent government regulation.^15^^,^^17^ For healthcare organizations, unlike the step-wise, systematic process for introducing new drugs into clinical practice,^18^ no equivalent approach exists for introducing AI interventions into hospitals. In contrast, researchers are developing, or have already released, standards for reporting studies relevant to each evolutionary stage of AI-based interventions, from retrospective evaluation of AI model performance (TRIPOD^2^^,^^19^; TRIPOD-AI^20^) through to prospective pilot evaluations (DECIDE-AI^21^) and large-scale clinical trials (CONSORT-AI^22^). These standards require researchers to fully disclose how they have developed and evaluated AI-based interventions. Integrating these standards within a clinical intervention implementation framework could provide a more systematic end-to-end clinical AI implementation framework, more akin to the process for introducing drugs that healthcare organizations are used to. In this paper we derive such a framework, intended for application within hospital care settings and to be used by a wide audience of stakeholders involved in developing, testing, deploying, funding, and governing AI-based decision support technologies.

### Background

We define implementation by extending the Cambridge dictionary definition^23^ as the act of starting to use a plan or system to change or incorporate a new intervention into clinical practice. We define AI as computer programs that learn from and can make predictions based on data, including machine learning/deep learning models. Theoretical clinical intervention implementation frameworks^24–28^ attempt to identify key stages, tasks, and contextual factors that warrant consideration. Nilsen defines a framework as, “a structure, overview, outline, system or plan consisting of various descriptive categories… and the relations between them that are presumed to account for a phenomenon.”^29^ An example of a parallel operational clinical implementation framework is the US Food and Drug Administration’s (FDA) Drug Development Process.^18^ Such a framework is needed in identifying clear steps and transparent evaluation gateways that provide a systematic pathway for organizations to minimize the risks associated with incorporating new drugs into clinical practice.

**Figure 1.**
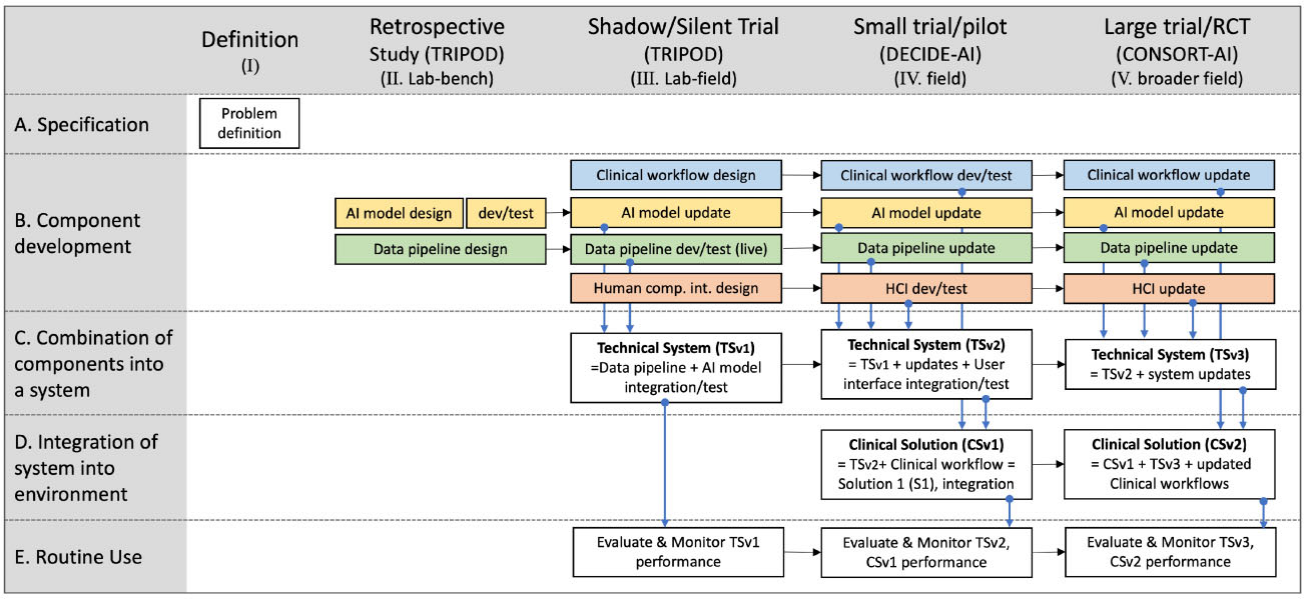
Provisional staged clinical AI implementation (SALIENT) framework. Adapted from Stead et al 12 and aligned with the TRIPOD, DECIDE-AI, and CONSORT-AI reporting guideline 39–42,44 stages and tasks. The colored boxes refer to solution components (see Element B). Blue for the clinical workflow, yellow for the AI model, green for the data pipeline, and red for the human computer interface. HCI: human computer interface; dev: development.

There is currently no equivalent widely acknowledged framework for implementing AI interventions into clinical practice, yet the systematic methodology to evaluate clinical AI implementation at multiple stages exists, as reported in Vasey et al’s Decide-AI reporting standard (Figure 1).^21^ Aligned to the serial stages (see Table 1) are the evaluation reporting standards of TRIPOD,^2^^,^^19^ TRIPOD-AI,^20^ DECIDE-AI,^21^ and CONSORT(-AI),^22^ herein referred to as the AI reporting standards. The standards are founded on long-serving, widely used (>10 000 citations) and effective^30^ intervention evaluation methodologies.^1^^,^^2^ Based on Gama et al’s review of existing theoretical AI implementation frameworks, none of the identified AI implementation frameworks explicitly integrate these standards.

**Table 1.** Translations of Stead et al’s levels of evaluation taxonomy to the research evaluation stage-based terminology.

Implementation stage	Stead et al’s stage (level of evaluation)	Research evaluation stage and associated reporting standard
I	I. Definition	Definition (from Stead et al)
II	II. Lab-bench	Retrospective study (TRIPOD)^2^^,^^19^
III	III. Lab-field	Shadow/silent study (TRIPOD)^2^^,^^19^
IV	IV. Field	Small trial/pilot (DECIDE-AI)^21^
V	V. Broader field	Large trial/RCT (CONSORT-AI)^22^^,^^43^

RCT: randomized controlled trial.

Prior clinical implementation frameworks generally fit into 2 of Nilsen’s 5 framework categories: Determinant and process models.^29^ Determinant frameworks identify themes or domains that can influence implementation outcomes, such as Greenhalgh et al’s nonadoption, abandonment, scale-up, spread, and sustainability (NASSS) framework.^27^ It has 7 key domains including clinical condition or context, technology, value proposition, adopters, organization, wider system, and embedding and adaption over time. It was derived through qualitative evaluation of technology implementation case studies complemented by a review of other frameworks. The NASSS framework and other similar conceptual or theme-based frameworks, such as the modified RE-AIM framework by Bakken et al,^26^ Damschroder et al’s Consolidated Framework For Implementation Research (CFIR)^25^ and Beil et al’s ethical pathway framework^31^ are positioned from a wide range of perspectives although none focus on AI implementation and most omit or remain unclear about the complete implementation cycle, including: (1) the start to finish staged sequence; (2) identification of key intervention components and associated tasks; and (3) progression and ultimate integration of all components into an end-to-end technical and clinical intervention.

Process models, which usually specify stages in the process of translating research into practice,^29^ redress some of these deficiencies. In the case of Sendak et al,^32^ a pathway consisting of 3 primary stages is proposed based on their experience: (1) design and develop; (2) evaluate and validate; (3) diffuse and scale. Van De Sande et al also proposed a step-by-step approach with 5 phases and 16 steps based on synthesis of data from a literature review.^33^ The phases are quite different to those of Sendak et al and others,^34^ and none are aligned with the AI reporting standards.

In summary, there are many implementation frameworks self-derived or derived from practice and prior literature that provide a wide range of differing perspectives and pathways for supporting healthcare organizations to implement AI. However, for the introduction of AI, as with the process for implementing other clinical interventions such as new drugs, staged evaluation of the intervention is central; yet none of the frameworks mentioned above are founded on this common approach. We hypothesized that by deriving a staged AI implementation framework directly aligned with the AI reporting standards and grounded in a well-established theory of translating clinical informatics interventions into practice, a more systematic staged approach would emerge. Because the AI reporting standards are limited by their focus on evaluation, we also sought to augment the derived framework with elements from prior AI frameworks.

### Objective

This study had 3 objectives: (1) Derive a provisional end-to-end clinical AI implementation framework that integrates the TRIPOD, DECIDE-AI, and CONSORT-AI reporting standards with an informatics translation theory; (2) Conduct a scoping review of clinical AI implementation framework studies to capture essential themes and stages; and (3) Refine the provisional framework by incorporating important missing elements identified from the scoping review.

## MATERIALS AND METHODS

### Derivation of a provisional clinical AI implementation framework

The AI reporting standards and associated item lists provide a foundation for a framework but lack process and structure. We therefore reviewed prior theoretical frameworks (AHV, IAS) for possible candidates that could align with the standards, identifying these through scrutinizing articles found within 3 review papers and further snowballing.^35–37^ We searched for frameworks that: (1) had similar stages to those of the AI reporting standards; and (2) were sufficiently flexible to support the development and implementation of AI solution elements derived from the AI reporting standards, which included the AI algorithm, data pipeline, human-computer interface, and clinical workflow. During candidate appraisal, determinant models were excluded because they did not support stages and would be radically modified by their addition.^25–28^^,^^38–41^ Process models did have stages,^33^^,^^42^ but they were fixed and varied in number and content from the AI reporting standards stages. Trying to retrofit a new set of stages would have violated those original frameworks. One exception was Stead et al’s taxonomy for translating medical informatics interventions from the laboratory to the field.^24^

Stead et al’s process framework considers how different components, technical and clinical, need to be developed and integrated, and in which of 5 evaluation stages these tasks need to occur: (I) Definition; (II) Laboratory—bench; (III) Laboratory—field; (IV) Remoter field—validity; and (V) Remoter field—efficacy. Stead et al identified 5 key elements in developing and implementing interventions: (A) Specification; (B) Component development; (C) Combination of components into a system; (D) Integration of system into environment; and (E) Routine use. We chose this as our baseline framework because of its clinical orientation, end-to-end nature, flexibility to incorporate solution components and close stage-alignment with the AI reporting standards.

While the stages of the Stead taxonomy are designed for any clinical informatics intervention, we intuited that analyzing the items in the current AI reporting standards may identify specific components and tasks that could be aligned with each stage for AI-based interventions. Accordingly, we used the following method to derive the provisional framework (see Supplementary Appendix SA for more details and examples):

Step 1: Define the baseline implementation stages: Map each implementation stage, as reported in the DECIDE-AI guideline,^21^ to the similar stage in the Stead taxonomy (see Table 1).

Step 2: Identify the intervention components and their associated implementation tasks: For each reporting item specified in TRIPOD, DECIDE-AI, and CONSORT-AI^19^^,^^21^^,^^22^^,^^43^ identify existing or create new components and component tasks and assign the implementation stage based on the mapping identified in Step 1, as exemplified in Table 2. This step was initially performed by AV and then using his draft task list, RS repeated the task creation independently. The final harmonized task set was agreed by consensus (RS, AV).

**Table 2.** Example of translating TRIPOD reporting item 9 to a component and component task.

TRIPOD report item 9:	*Describe how missing data were handled (eg, complete-case analysis, single imputation, and multiple imputation) with details of any imputation method*
Task(s) created:	Define and handle missing data (imputation)
Component created:	Data pipeline
Stage:	Retrospective and silent tracking

Step 3: Consolidate similar components and component tasks identified in Step 2 into a final reduced task and component set.

Step 4: Back- or forward-fill missing tasks across stages as, in some instances, tasks are identified in one stage and required in earlier or later stages but the latter stage reporting standards make no provision for them. For example, CONSORT-AI item 4b (Extension) is, “Describe how the AI intervention was integrated into the trial setting, including any onsite or offsite requirements.”^22^ A stage V task generated from this item is the data pipeline component task, “Develop real-time data capture/transform capability.” However, this task is required in both the silent study stage (III) and the small pilot trial stage (IV), where real-time data are also required, and hence this task is copied backwards across the earlier stages.

Step 5: Identify the components that make up technical systems (TS) and clinical solutions (CS) at each stage (I to V) which apply to Stead et al’s elements C (Combination of components into a system) and D (Integration of system into environment). Finally, element E (Routine use) incorporates the evaluation and performance monitoring tasks for both the technical system and overall clinical solution.

### Scoping review of clinical AI implementation framework studies

The scoping review consisted of a comprehensive systematic search for existing AI implementation frameworks, with analysis limited to identification of themes and stages reported in the identified frameworks.^44^ It was reported according to the PRISMA Extension for Scoping Review (PRISMA-ScR) guidelines.^45^ No formal quality assessment of the papers was performed, although the source and derivation of the frameworks were reported.

#### Search strategy

Five databases (Pubmed/Medline, EMBASE, Web of Science, CINAHL, and IEEExplore) were searched up to November 25, 2022 for titles and abstracts published in English using keywords and synonyms for: (1) AI or “artificial intelligence” or “machine learning”; AND (2) framework or “step-by-step” or roadmap; AND (3) implement* or deploy* or adopt*. For nonclinical databases, a “medic* OR clinic*” search phrase was appended with an AND statement (See Supplementary Appendix SB for complete search queries).

#### Study selection

All studies proposing a framework for implementing AI into clinical practice were included unless solely focused on imaging applications or a single AI solution, eg, a specific information technology (IT) infrastructure or a specific clinical task, such as sepsis prediction (full eligibility details in Supplementary Appendix SC). Covidence software^46^ supported a 2-stage screening process: (1) Screening of abstracts and titles by 3 independent reviewers (AHV, PL, or VK) with conflicts agreed by 3-way consensus (AHV, VK, KD); and (2) Full-text review conducted by 2 independent reviewers (AHV, KD), with selection agreed by 3-way consensus (AHV, RJS, KD).

#### Data extraction

Data from each paper were extracted into an Excel template and comprised study metadata, objective, clinical setting, theoretical underpinnings, methods for deriving the framework, and details relating to themes and stages. (See Supplementary Appendix SD for listing of data elements extracted).

### Refinement of provisional implementation framework

A gap analysis was performed (AV, KD) to identify lack of concordance between the themes and stages extracted from each paper in the review and the stages, components and tasks of the provisional framework (see Supplementary Appendix SE for further mapping details). Missing or partially mapped elements were grouped and assigned to one of: (1) new stage; (2) new cross-stage element, where the missing element was applicable across more than one stage; (3) new component; or (4) new component task. The purpose of this step was to augment SALIENT with prior framework themes and stages that it was missing, generating a more comprehensive and useful final AI implementation framework.

## RESULTS

### Derivation of provisional implementation framework

The outputs of the 5-step process were used to derive our provisional implementation framework, titled the staged clinical AI implementation (SALIENT) framework (Figure 1). This comprised 5 implementation stages, labeled I to V, positioned across the top of Figure 1 and 5 elements, labeled A to E, positioned down the left-hand side. Element A, specification, describes preparatory work to clearly articulate the problem definition and proposed intervention (hereafter termed solution) specification. Element B comprises the development of 4 essential solution components: (1) AI model; (2) data pipeline; (3) human-computer interface (HCI); and (4) clinical workflow. Component development is divided into 3 engineering steps: (1) design; (2) develop and test; and (3) update. The stage-timing of these steps depends on the solution requirements at each stage for each component. For example, at the retrospective stage (II), the AI model is designed, developed, and validated using static datasets, whereas development and testing of the data pipeline using live or near-live data is only required at the silent study stage (III).

Element C of SALIENT combines solution components into functioning systems over 3 stages. Firstly, the technical system (TSv1) comprising the AI model and data pipeline are integrated for the silent study stage (III). In Stage IV, the HCI must also be integrated (TSv2) so that evaluation of clinician-computer interactions can be performed. Following further iterations and refinement of the system in response to these study results, the final technical system is completed for the large trial or roll-out in Stage V. Element D of SALIENT marks the coming together of the overall solution when the system is integrated into the live, routine clinical practice environment. The clinical solution (CSv1) must be ready at stage IV, comprising the technical solution (TSv2) and the clinical workflows, which is then updated ready for final trial and rollout in stage V. Element E, routine use, denotes all tasks required for normal continuous operation of the solution.

Table 3 identifies the components and tasks across each element (A to E) in the framework. For example, there are 4 tasks (AM01-04) itemized for the AI model component within element B (Component development) and 11 tasks identified for Element E (routine use). The individual reporting standard items (TRIPOD, DECIDE-AI, CONSORT-AI) accounting for each component task are specified in the respective, color-coded stage column (II/III [pink], IV [grey] and V [purple]). As previously noted, sometimes a task is needed in an earlier or later stage, but a relevant reporting item is missing in that stage. Where a task has been copied backward to an earlier stage (denoted by ‡) or copied forward to a later stage (denoted by §), the originating stage color is preserved in the earlier or later stage cell in the table so that one can see from which stage the task was derived.

**Table 3.** Implementation tasks (left hand column) mapped to each reporting guideline item (right-hand 3 columns) and allocated to the provisional SALIENT AI framework elements (A, B, C, D, E) and components.

SALIENT framework: components and tasks		TRIPOD Stages II/III	DECIDE—AI Stage IV	CONSORT-AI Stage V
**Framework element A: specifications**				
**Component: problem definition (PD)**				
P1	Rationale for change, background, context	3a	2	2a; 2a(i)
P2	Intended use		2	2a; 2a(i)
**Framework element B: component development**				
**Component: artificial intelligence model (AM)**				
AM01	Select and define data elements (predictors) required and units	7a; 15a	4b	5(ii)
AM02	Select/develop AI model(s) versions (including comparators) and internally validate; specify how predictions are calculated	10b; 10c; 15a	4a	5(i)]
AM03	Define and implement model calibration/fine tuning process	10d	§	4b(ext)
AM04	Define and execute AI model update process and code management	10e	11; 17	5(i); 25(ext)
**Component: data pipeline (DP)**				
DP01	Identify input data capture method (automated/manual) Clarify/specify measurement methods/units for capture	7a	4b	^§^
DP02	Identify source systems for data elements	^‡^	4b	^§^
DP03	Add/extend systems for any new data element entry	^‡^	4b	^§^
DP04	Identify eligible patients: inclusion/exclusion criteria + minimum data requirements/patient level sample size	5b; 8	3a; 9a	4a(ii); 7a; 14a
DP05	Identify and select data elements required	5c; 7a	4b	5(ii)
DP06	Transform data: Clean poor quality data (eg, outlier/invalid data)/Define and handle missing data (imputation)/Define and handle feature/predictor transformations/Perform other necessary data preprocessing steps	9; 10a; 7a	4b; 9a	5(ii); 5(iii)
DP07	Develop end-to-end data-pipeline for: AI models, comparative models, patient outcome, and risk group identification	6a; 7a; 11	4b; IV	6a; 6b
DP08	Develop real-time data capture/transform capability	^‡^	^‡^	4b(ext)
DP09	Build or procure retrospective data set for AI training/validation/test	4a	4a	
DP10	Define and execute pipeline update process and code management	^‡^	11; 17	^§^
**Component: human-computer interface (HCI)**				
HC1	Define and develop content, layout and format of interface for users, including level of user customization	^‡^	4c	^§^
HC2	Specify update frequency, design, develop, and test real-time HC interface system	^‡^	4c	4b(ext); 5(iv); 5(v); 9
HC3	Conduct preclinical human factors evaluation		7	
HC4	Define and execute HC interface update process and code management	^‡^	11; 17	^§^
**Component: clinical workflows (CW)**				
CW01	Identify target patients: eligibility, location(s), settings, sample sizes	4b; 5a; 5b; 5c; 8	2a; 3a	4a; 4a(i); 4b; 14a; 7a
CW02	Identify target patient outcomes and subgroup definitions	6a; 11	IV	6a; 6b
CW03	Identify participant clinicians (users)	^‡^	3b; 9b	^§^
CW04	Reengineer clinical workflow/care pathways to include AI information; Clarify/specify decision making process incorporating the AI; Establish user agreement with AI system		2b; 5a; 5b; 10b; 12	4b(ext); 5(vi)
CW05	Develop, test, execute processes to capture failures and feedback: user errors and feedback, software/AI malfunctions, patient harms/risks	^‡^	6a; 13a; 13b	5(vi); 19(ext); 19
CW06	Develop, test, and execute staff training in new process and AI system training	^‡^	3c	5(iv)
CW07	Conduct pre/postclinical risk-assessment and mitigation plan		6b; 16	
**Framework element C: combination of components into a system**				
**Technical system (TS)**				
TS01	Integrate data pipeline with AI model output	^‡^	^‡^	4b(ext)
TS02	Integrate human-computer interface and changes to AI model/data pipeline		4c	4b(ext); 5(iv); 5(v); 9
TS03	Integrate any changes from human-computer interface/AI model/data pipeline		11; 17	5(i); 25(ext)
**Framework element D: integration of system into environment**				
**Clinical solution (CS)**				
CS01	Integrate clinical workflows with technical system (TS02)		2b; 5a; 5b; 10b; 12	4b(ext); 5(vi)
CS02	Integrate updates to clinical workflows and the technical system (TS03)		2b; 5a; 5b; 10b; 12	4b(ext); 5(vi)
**Framework element E: routine use**				
**Evaluation and monitoring (EM)**				
EM1	Select models for comparative evaluation	3a		
EM2	Report patient population characteristics (dataset shift) including treatments received; also report patient flow, exclusions and outcome/risk group results	4a; 5c; 11; 13a; 13b	4a; 9a	12b; 13; 13a; 15; 16
EM3	Define and report metrics for AI model validation, evaluation and comparison and any updated models	10d; 16; 17	^§^	^§^
EM4	Define and report errors: user, system malfunctions and AI model errors, safety	^‡^	6a; 10a; 13	19(ext)
EM5	Define and report AI system safety evaluation, harm and unintended effects	^‡^	6b; 13a	19
EM6	Define and report HCI usage, use-variation and usability evaluation (eg, NASA’s Task load index^47^)		7; 10a; 13a, 14a; 14b	5(vi)
EM7	Define and report AI system biases	^‡^	8	20
EM8	Define and report data missingness	10a; 13b	9a	^§^
EM9	Define and report clinical results (outcomes by subgroup)		V; VII; VIII	12a; 13; 13a; 17a; 17b
EM10	Report deeper analyses of results (by outcome), eg, univariate associations	14a; 14b	^§^	12b; 18
EM11	Report differences to prior phase (setting, outcome, predictors, results)	12; 13c; 19a	^§^	^§^

Note that the reporting guideline items are referenced exactly as they appear in the guideline papers and can be alpha-numeric (eg, 8, or 9a), Roman numerals (eg, VII, 5[vi]) and extensions, denoted “ext.” Each SALIENT stage is color-coded: White means no guideline element is applicable; pink for retrospective and silent trial stages II and III (TRIPOD^40^^,^^44^); grey for pilot/trial stage IV (DECIDE-AI^39^); purple for large-trial/roll-out stage V (CONSORT-AI^41^^,^^42^).

‡ Task missing: copy task backward from later project stage.

§ Task missing: copy task forward from previous project stage. See text for more details.

### Scoping review of clinical AI implementation framework studies

**Figure 2.**
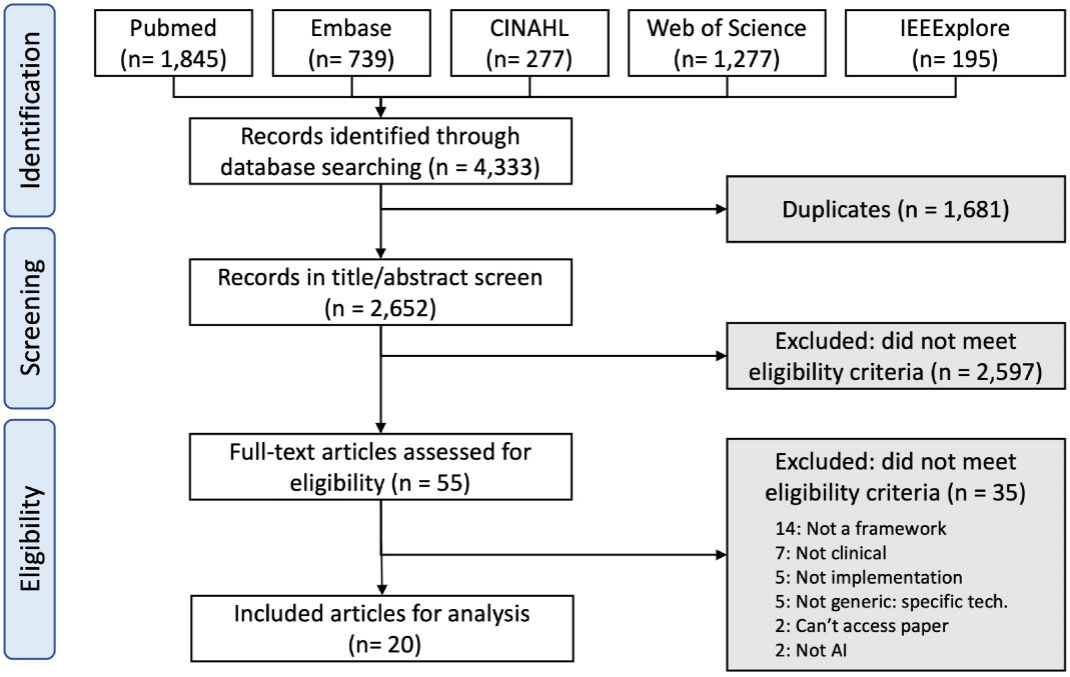
PRISMA-ScR flowchart for study selection.

From 4333 retrieved abstracts, 1681 duplicates were removed, leaving 2652 for screening from which 20 full-text articles^19–38^ were included for analysis (Figure 2).

#### Study characteristics

All 20 studies were published between 2019 and 2022, with 70% (*n* = 14) published in 2021–22 (see Table 4). Ten studies were from the United States, 3 from Canada, 2 from the Netherlands, 2 from Europe and one each from Australia, United Kingdom, and Sweden. Half of the studies (*n* = 10) were frameworks targeting specific domains: (1) specific clinical disciplines including oncology,^48^^,^^49^ radiology (but not entirely focused on imaging processing or interpretation),^50^ or paediatrics^51^; (2) evaluation^52^^,^^53^; (3) ethics^48^^,^^54^; (4) governance^50^^,^^55^; (5) regulation^56^; and (6) safety.^57^ Of the other 10 generic framework papers, 5 were process frameworks (stage-based)^33^^,^^42^^,^^58–60^ with 3 to 7 (median 5) stages, and 5 were determinant frameworks (theme-based) with 3 to 7 themes (median 5).^35^^,^^38^^,^^61–63^

**Table 4.** Study characteristics.

Author, year, country		Source of framework, stage/dimensions identified
Stage-based (process) frameworks:		
1	van de Sande et al 2022,^33^ Netherlands	Source: Literature review; author creation.
		Phases: (1) Preparation; (2) Model development; (3) Assessment of AI performance and reliability; (4) Clinically testing AI; (5) Implementing and governing of AI.
		16 substeps identified
2	de Hond et al 2022,^59^ Netherlands (AIPM)	Source: Multiple prior models + scoping review (72 papers).
		Phases: (1) Preparation, collection and checking of the data; (2) Development of the AIPM; (3) Validation of the AIPM; (4) Development of the software application; (5) Impact assessment of the AIPM with software; (6) Implementation and use in daily healthcare practice.
		27 substage steps and a further 6 phase overarching topics.
3	Sendak et al 2020,^42^ United States	Source: Prior models^58^^,^^64^ + own experience.
		Phases: 3 unnamed.
		8 substeps
4	Wiens et al 2019,^58^ United States	Source: Author created.
		Phases: (1) Choosing the right problem; (2) Developing a useful solution; (3) Considering ethical implications; (4) Rigorously evaluating the model; (5) Reporting results; (6) Deploying responsibly; (7) Making it to market
5	Assadi et al 2022,^60^ Canada	Source: Narrative review and expert consensus, based on systems engineering and software development.
		Phases: (1) Inception; (2) Preparation; (3) Development; (4) Integration.
		3 substeps (technical systems, human, environment) identified per phase.
Theme-based (determinant) frameworks:		
1	Gama et al 2020,^35^ Sweden	Source: Scoping review (7 papers), derived from NASSS.^27^
		Themes: Same as NASSS: (1) Condition or illness; (2) Technology; (3) Value proposition; (4) Adopter system; (5) Organization(s); (6) Wider context; (7) Interaction and mutual adaptation between domains.
		22 subdomains and 7 new subdomains
2	Truong et al 2019,^38^ Canada	Source: Meetings with subject matter experts from 4 hospitals.
		Themes: (1) Data; (2) Trust; (3) Ethics; (4) Readiness; (5) Expertise; (6) Buy-in; (7) Regulatory strategy.
3	Salwei et al 2022,^61^ United States	Source: Self-created and non-AI case study.
		Themes: (1) Integrate AI into work system; (2) Integrate AI into clinical workflow; (3) Support decision making.
4	Oala et al 2021,^62^ Europe	Source: Consensus findings, self-created.
		Themes: (1) Technical validation; (2) Clinical evaluation; (3) Regulatory assessment
5	Siala and Wang, 2022,^63^ United Kingdom (SHIFT-AI)	Source: Literature review (253 papers) and inductive thematic analysis.
		Themes; (1) Sustainable AI; (2) Human-centered AI; (3) Inclusive AI; (4) FAIR AI; (5) Transparent AI.
		14 subthemes identified
Targeted frameworks:		
1	Park et al 2020,^52^ United States	Targeting: Evaluating AI in healthcare.
		Source: Mapping of study designs to drug/medial trial phases.
		Phases: (1) Discovery and invention; (2) Safety and dosage; (3) Efficacy and side effects; (4) Therapeutic efficacy; (5) Safety and effectiveness.
2	Reddy et al 2021 (TEHAI),^53^ Australia	Targeting: Evaluating real-world artificial intelligence systems.
		Source: Literature review (6 papers) and consensus inclusion with panel. Components: (1) Capability; (2) Utility; (3) Adoption.
		15 subcomponents
3	Hantel et al 2022 (A4R-OAI),^48^ United States	Targeting: Ethically deployed oncology AI.
		Source: Based on accountability for reasonableness framework.
		Principles: (1) Relevance; (2) Publicity; (3) Revision; (4) Empowerment; (5) Enforcement.
4	Bedoya et al 2022 (ABCDS),^55^ United States	Targeting: Governance.
		Source: Software development cycle, FDA regulatory best practice and self-created and implemented governance methodology.
		Phases: (1) Model development; (2) Silent evaluation; (3) Effectiveness evaluation; (4) General deployment.
		Further 16 subphase steps.
5	Bazoukis et al 2022,^56^ United States	Targeting: Integrated regulatory framework.
		Source: Self-created.
		Themes: (1) Regulatory challenges; (2) Oversight and regulation; (3) Safety and efficacy surveillance; (4) Accountability; (5) Liability; (6) Equity and inclusion; (7) Transparency; (8) Education; (9) Patient engagement; (10) Cybersecurity and privacy; (11) Ethics and fairness; (12) Financial incentives.
6	Char et al 2020,^54^ United States	Targeting: Ethical AI implementation.
		Source: Literature review (83 papers) and author created.
		Stages: (1) Conception; (2) Development; (3) Calibration; (4) Initial implementation; (5) Subsequent implementations.
7	Davahli et al 2021,^57^ United States	Targeting: Safety control system framework.
		Source: Multiattribute value model approach, systematic review (67 papers), 10 interviews, 2 surveys.
		Domains: (1) Safety policy; (2) Incentives for clinicians; (3) Clinician and patient training; (4) Communication and interaction; (5) Planning of actions; (6) Control on actions.
		13 second level attributes.
8	Nagaraj et al 2020,^51^ Canada	Targeting: Pediatric care.
		Source: Self-created.
		Stages: (1) Clinical use-case design; (2) Data acquisition and preparation; (3) Model development; (4) Model validation; (5) User validation; (6) Clinical integration; (7) Legal, privacy, and ethical considerations.
		5 further substeps.
9	Daye et al 2022,^50^ United States	Targeting: Radiology/governance.
		Source: Self-created.
		Steps: (1) Who decides which tool to implement; (2) What should be considered when assessing a tool for implementation; (3) How should each application be implemented in clinical practice; (4) How should tools be monitored and maintained for implementation.
		Step 3 has 3 substeps.
10	Tsopra et al 2021,^49^ Europe	Targeting: Clinical validation of AI technologies for prediction in oncology.
		Source: The ITFoC (Information Technology for the Future of Cancer) consortium, a multidisciplinary group from 6 European countries, self-created.
		Principles: (1) Specify the intended use of AI; (2) Clearly specify the target population; (3) Specify the timing of AI evaluation; (4) Specify the datasets used for AI evaluation; (5) Specify the procedures used to ensure data safety; (6) Specify the metrics used for measuring AI performance; (7) Specify the procedures to ensure AI explainability.

Framework names in parenthesis, if provided.

Five studies proposed frameworks without a stated methodology,^50^^,^^51^^,^^56^^,^^58^^,^^61^ 8 utilized literature reviews,^33^^,^^35^^,^^53^^,^^54^^,^^57^^,^^59^^,^^60^^,^^63^ and 7 derived their framework from prior frameworks.^35^^,^^48^^,^^52^^,^^55^^,^^57^^,^^59^^,^^60^ Four studies employed a consensus method,^49^^,^^53^^,^^60^^,^^62^ 2 used subject matter expert interviews,^38^^,^^57^ and 2 were based on authors’ own experiences.^42^^,^^55^ Two studies utilized 3 of the above methods^57^^,^^60^ and 4 studies utilized 2.^35^^,^^53^^,^^55^^,^^59^ Given the recency of all frameworks, assessing study acceptance using citation counts was not performed.

### Refinement of the provisional SALIENT framework

Of the 247 stages and themes (including subelements) extracted from the 20 included papers, 37% (*n* = 92) could be fully mapped to the provisional SALIENT framework, 40% (*n* = 98) could be partially mapped and 23% (*n* = 57) could not be mapped at all (see Supplementary Appendix SE for complete mapping). The gap analysis consolidated the partial and unmapped elements and informed the inclusion into SALIENT of 5 new cross-stage themes, 3 Stage I (Definition) component additions, and 16 new component tasks.

Two of the cross-stage themes—(1) Implementation, change management, and adoption^33^^,^^38^^,^^42^^,^^48^^,^^51^^,^^53^^,^^56^^,^^58–60^; and (2) Governance^33^^,^^42^^,^^50^^,^^53^^,^^56^—were housed in a new SALIENT element, “F. Organisation engagement.” These 2 cross-stage themes were informed by prior framework findings. Implementation, change management, and adoption required (1) the clear identification and engagement of all stakeholders, including not just clinicians and data scientists, but patients, ethicists, social scientists, managers, and legal experts^33^^,^^38^^,^^48^^,^^51^^,^^56^^,^^58–60^; (2) use of broad communication strategies, especially regarding stakeholder roles and responsibilities^38^^,^^42^^,^^51^; and (3) planning to generate long-term clinical buy-in and adoption, especially for nondevelopment sites with possibly different clinical workflows.^38^^,^^42^^,^^51^^,^^53^^,^^59^ The second cross-stage theme, Governance, involves the arrangements for providing program oversight, deciding on final AI model selection, timing and readiness for implementation, and ensuring various governance standards (see below), are known and upheld.^33^^,^^42^^,^^50^^,^^53^^,^^56^

Three other cross-stage themes were grouped into a new SALIENT element, “G. Policy domains,” comprising (1) Regulatory and legal^33^^,^^38^^,^^48^^,^^51^^,^^56^^,^^57^^,^^60^; (2) Ethics, including privacy, transparency, and equity^33^^,^^38^^,^^48^^,^^49^^,^^51^^,^^54–60^; and (3) Quality and safety.^33^^,^^38^^,^^50^^,^^54^^,^^56–59^ The first domain is awareness by all concerned of the relevant jurisdictional legal and regulatory evaluation and approval frameworks prior to AI implementation.^33^^,^^38^^,^^48^^,^^56^^,^^57^^,^^60^ Healthcare organizations and their clinicians need to understand who assumes liability and accountability for using AI model outputs in making clinical decisions.^51^^,^^56^^,^^57^ The second domain of ethics has 3 components: (1) Data privacy, including compliance with privacy laws, mandating consideration of data ownership, data traceability, right to privacy, and cyber security protections to prevent breaches^33^^,^^38^^,^^48^^,^^49^^,^^51^^,^^53^^,^^54^^,^^56^^,^^58–60^; (2) Transparency in relation to who generates the AI model and who uses model outputs, including the degree of clinician autonomy (assistive or autonomous AI), locked or adaptive (continuously learning) AI model,^38^^,^^48^^,^^54^ and scope,^33^^,^^38^^,^^57–59^ interpretability and auditability^38^^,^^48^^,^^49^^,^^53^^,^^54^^,^^59^ of the AI model; and (3) Healthcare equity including assessments and monitoring of model fairness and bias across all stages to protect minority populations.^38^^,^^51^^,^^53–56^^,^^58–60^ The third domain of quality and safety includes: (1) automated systems to detect data shift and where necessary retire, retrain or upgrade AI models^33^^,^^50^^,^^54^^,^^58^^,^^59^; (2) quality management systems to monitor for clinical practice updates that might disrupt AI model inputs or corrupt AI model accuracy; (3) systems for logging and tracing clinician decisions in response to model outputs^59^; (4) risk management strategies and safety surveillance for capturing adverse events related to AI-based decisions and determining agreed accuracy thresholds for the timely recall of AI models if becoming unreliable^30^^,^^53^^,^^56^^,^^57^; and (5) safety incentive programs to promote the judicial use by clinicians of AI rather than blind reliance.^57^

**Figure 3.**
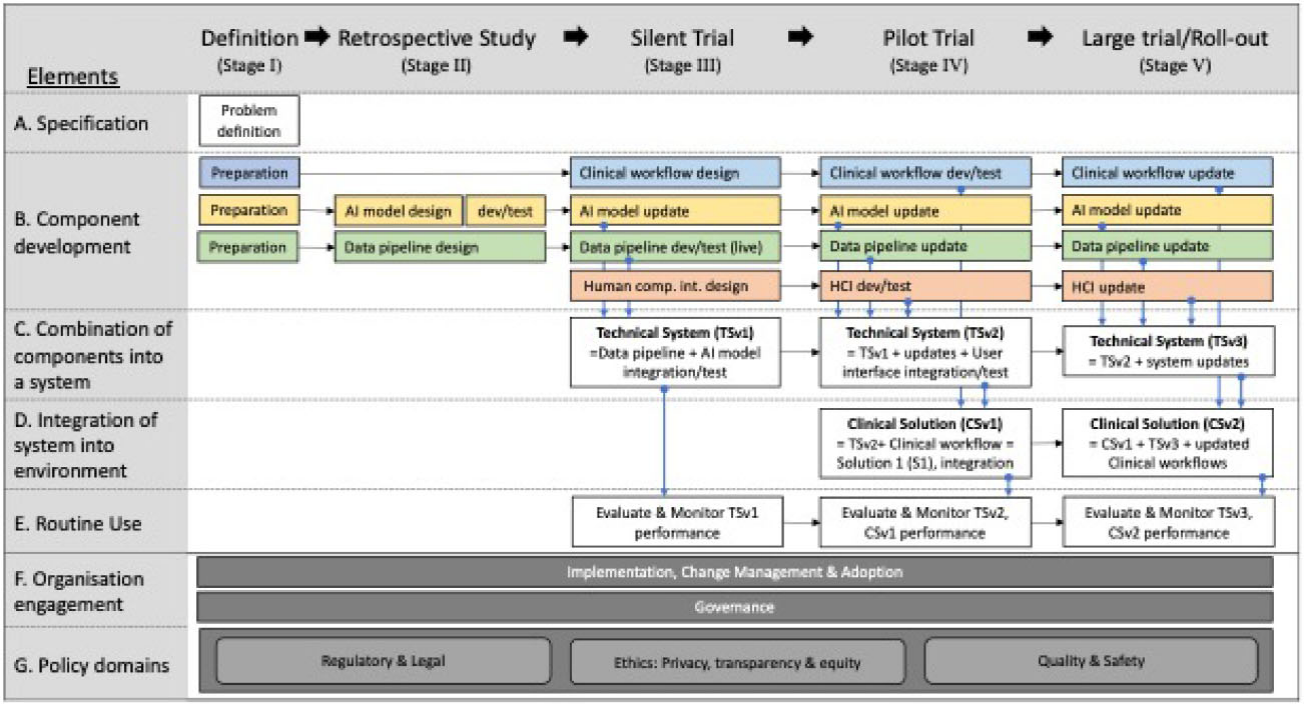
Final clinical AI implementation framework (SALIENT). The colored boxes refer to solution components (see Element B). Blue for the clinical workflow, yellow for the AI model, green for the data pipeline, and red for the human computer interface. The dark grey shaded boxes identify the cross-stage elements (F and G). HCI: human computer interface; dev: development.

The stage I definition element was also expanded to include 6 preparation tasks for the AI model, clinical workflow and data pipeline components, 10 other new tasks were integrated into existing components, and all tasks, respective sources and applicable stages are shown in Table 5, with the finalized SALIENT framework depicted in Figure 3.

**Table 5.** New tasks informed by findings from the scoping review of prior AI implementation frameworks, grouped by the SALIENT AI framework component (column 1, blue text).

Revised SALIENT framework: components and tasks		Stages II and III	Stage IV	Stage V
**Framework element A: specifications**				
**Preparation—data pipeline**				
PDP01	Identify, collect, and prepare data for development and validation, including training data^54^^,^^58–60^			
PDP02	Establish interoperability and align to clinical coding standards (eg, ICD codes)^33^^,^^49^^,^^59^			
PDP03	Investigate and establish IT hardware and storage capability^60^			
**Preparation—AI model**				
PAM01	Search for and evaluate existing AI models^33^^,^^42^			
PAM02	Perform a cost benefit analysis and feasibility assessment of using AI^27^^,^^35^^,^^53^^,^^59^			
**Preparation—clinical workflow**				
PCW01	Create a plan to evaluate the success of the implementation^38^^,^^53^			
**Framework element B: component development**				
**Data pipeline (DP)**				
DP11	Stress test infrastructure^59^	x	x	x
DP12	Scalability assessment: assess changes to data sources and input protocols^27^^,^^35^		x	x
**Artificial intelligence model (AM)**				
AM05	Devise means and document interpretability of AI model outputs^38^^,^^48^^,^^49^^,^^53^^,^^59^	x	x	
AM06	Define, publish, and update AI model fact label defining standardized communication of AI model information to end users^33^^,^^38^^,^^57–59^	x	x	x
AM07	Externally validate the model and assess generalizability^33^^,^^49^^,^^53^^,^^58^^,^^59^	x	x	
AM08	Check for, report on, and apply methods to reduce overfitting^59^	x	x	
AM09	Stress test AI model software^59^		x	
**Human-computer interface (HC)**				
HC5	Stress test HC interface software^59^		x	
**Framework element E: routine use**				
**Evaluation and monitoring**				
EM12	Monitor and track data shift and AI quality^27^^,^^33^^,^^35^^,^^50^^,^^53^^,^^54^^,^^58^^,^^59^			x
EM13	Log AI decisions for traceability^59^		x	x

Applicable stages for each task, according to the TRIPOD, DECIDE-AI, and CONSORT-AI defined stages, are marked with an “x” in the appropriate columns, ie, for stage II/III (blue column), stage IV (amber column), and stage V (green column). Note that preparation tasks (PDP, PAM, PCW) are only applicable to stage I (Definition), which is not marked here.

ICD: International Classification of Diseases; IT: information technology; AI: artificial intelligence.

## DISCUSSION

Our overarching aim was to develop a comprehensive, end-to-end clinical AI implementation framework that was integrated with current reporting standards for clinical AI research and informed by contemporary theories of staged AI implementation. Existing stage and theme-based frameworks were deemed inadequate in demarcating solution components or associated tasks and none incorporated the reporting standards. Our pragmatic staged approach, adapted from Stead et al, sought to fill this gap by addressing the what (components), when (stages), and how (tasks) of AI implementation, while the who (organization) and why (policy domains) were captured through the 5 cross-stage elements. In this way, SALIENT encompasses the “organisation,” “adopters,” and “wider systems domains” of the NASSS framework,^27^ the process implementation domain of the CFIR,^25^ and the “ethics,” “buy-in,” and “regulatory strategy” themes of Beil et al^31^ and Truong et al.^38^

The fact that 70% of AI implementation studies in our scoping review appeared within the last 2 years, with no studies prior to 2019, suggests AI framework theory has lagged behind the early adopters who deployed AI systems prior to 2019^65–68^ and had to confront new challenges unaided by a fully developed implementation framework.^7^^,^^69^^,^^70^ Many of the subsequent frameworks found in our review were informed by these early experiences^48^^,^^56^^,^^60^^,^^61^^,^^63^ and a quarter specifically targeted emerging areas of common concern, including regulatory requirements,^56^ ethical concerns,^48^^,^^54^ and governance,^50^^,^^55^ which were captured in the new SALIENT cross-stage elements F and G.

The SALIENT framework is unique in several ways. Firstly, it includes both theme and stage elements, whereas all frameworks except one^59^ are either process or determinant. Secondly, SALIENT stands alone in mapping and integrating all elements of the reporting standards applicable to studies of AI development and evaluation. van de Sande et al^33^ and de Hond et al^59^ integrated some elements of these standards, and some are mentioned in 3 other frameworks.^50^^,^^53^^,^^61^ Crossnohere et al assessed the coverage of 14 descriptive and reporting clinical AI implementation frameworks across 5 content domains (transparency, reproducibility, ethics, effectiveness, and engagement) and showed CONSORT-AI and DECIDE-AI together covered 17 of 25 (68%) content items.^71^ By integrating these reporting standards, clinicians can be assured that AI implementation based on SALIENT is grounded in rigorous evaluation methodologies. Thirdly, by adapting Stead et al’s clinical informatics translation approach, SALIENT provides full visibility of the end-to-end solution scope including its intrinsic components, how and when they integrate, and the underlying implementation tasks.

This stand-alone implementation framework study has an associated companion study^72^ in which the utility of the SALIENT framework is validated by applying it to studies of deployed AI models for predicting sepsis in hospitalized patients, identified in a systematic review, and mapping the barriers, facilitators and key implementation decisions reported in these studies to the SALIENT framework. This companion study found that SALIENT had full coverage of all the stages and components of implementing sepsis AI prediction systems which need to be considered and accounted for.

### Strengths and limitations

As far as we know, SALIENT is the only clinical AI implementation framework that conceptualizes all important tasks and solution components as one integrated schema (Figure 3). It provides immediately actionable insights, in for the form of checklists of component tasks for each implementation stage, for both AI developers and healthcare leaders wanting to successfully deploy clinical AI in real time and at a whole-of-organization level. SALIENT allows both clinicians and technologists to drill down, with a high level of structured detail missing in other guidance reports,^30^ to task-level responsibilities for each stage of implementation and for each component of the overall AI solution.

SALIENT is limited in that it attempts to present a generalizable and purpose-agnostic conceptualization of real-world AI implementation. Consequently, it cannot provide high-level granular detail for each task and theme relevant to specific AI applications, although each theme is extensively cited with primary sources that provide more information about specific areas of regulatory compliance,^56^ ethical concerns,^48^^,^^54^ governance,^50^^,^^55^ and patient and public involvement,^73^^,^^74^ all of which may vary across different jurisdictions.^75^ While SALIENT has been mapped to systematically retrieved studies of implemented sepsis prediction models (see companion paper), it requires further validation as a framework capable of meaningful application to real-world studies of deployed purpose-specific AI models.

## CONCLUSIONS

This study has generated a novel end-to-end framework for implementing clinical AI within hospitals which has integrated existing theoretical frameworks with current reporting standards for research related to AI models. Its use may help healthcare organizations to navigate the steps required to successfully implement AI in clinical practice.

## Supplementary Material

ocad088_Supplementary_Data

## Data Availability

There are no new data associated with this article.
