# Supplementary material for: Implementation frameworks for end-to-end clinical AI: derivation of the SALIENT framework
Source: J Am Med Inform Assoc. 2023 May 19;30(9):1503–15. doi: 10.1093/jamia/ocad088 (PMC10436156; doi:10.1093/jamia/ocad088)
Supplement: ocad088_Supplementary_Data [file ocad088_supplementary_data.docx]

Supplementary Appendices

Table of Contents

[Appendix A: Method for Integrating stage tasks and Stead et al.’s components into derived framework 2](#_Toc122533033)

[Appendix B: Search Queries 4](#_Toc122533034)

[Pubmed Search Query 4](#_Toc122533035)

[EMBASE Search Query 4](#_Toc122533036)

[Web of Science Search Query 4](#_Toc122533037)

[CINAHL Search Query 5](#_Toc122533038)

[IEEExplore Search Query 6](#_Toc122533039)

[Appendix C: Eligibility Criteria 6](#_Toc122533040)

[Inclusion criteria: 6](#_Toc122533041)

[Exclusion criteria: 6](#_Toc122533042)

[Appendix D. Extracted data items 7](#_Toc122533043)

[Appendix E: Mapping Review Paper Stages/Themes to Provisional Framework 8](#_Toc122533044)

[Table E1 (over 5 pages) : Review paper theme mapping to provisional framework tasks 9](#_Toc122533045)

[Table E2 (over 4 pages) : Review paper stage mapping to provisional framework tasks 13](#_Toc122533046)

[Table E3: Summary of Mapping 18](#_Toc122533047)

# Appendix A: Method for Integrating stage tasks and stead et al.’s components into derived framework

The following method is used to derive each element of the proposed framework:

1. Define the implementation stages: Map each reporting guideline stage, depicted in Vasey et al. to the similar stage in Stead et al.’s work.
2. Identify the solution components and component tasks: For each reporting item listed in the TRIPOD, DECIDE-AI and CONSORT-AI reporting guidelines and explanatory notes: ^39–42,44^
   1. Identify the essential solution component (Stead et al.’s components) that the item relates to
   2. Identify an implementation task that relates to the component
   3. Assign the implementation stage based on the mapping identified in (1)
3. Further consolidate the tasks identified in (2b) into fewer, common tasks and identify combined system and solution components
4. Identify the combination of components that make up technical systems and clinical solutions at each stage

For step (1), the resulting mapping is shown in supplementary Table A1 and is reflected in the top heading line of Figure 1. Our given name for each stage is shown, plus the associated reporting guideline and mapped column title from Stead et al. , Table 1 (depicted in parentheses). ^12^

| **Implementation Stage** | **Stead et al.’s (Table 1)^12^ stage** | **Given stage name and associated Reporting Guideline** |
| --- | --- | --- |
| I | I. Definition | None |
| II | II. Lab-bench | Retrospective Study (TRIPOD)^29^ |
| III | III. Lab-field | Shadow/Silent Trial (TRIPOD)^29^ |
| IV | IV. Field | Small trial/pilot (DECIDE-AI)^28^ |
| V | V. Broader field | Large trial/RCT (CONSORT-AI)^30^ |

Table A1: Mapping of Stead et al (Table 1) stages to the equivalent reporting guidelines^28, 29, 30,^ stages.

For step (2), An example of this mapping is provided below in Table D2 for the Tripod reporting item 9.

| TRIPOD report item 9 | Describe how missing data were handled (e.g. complete-case analysis, single imputation and multiple imputation) with details of any imputation method |
| --- | --- |
| Task(s) created: | Define & handle missing data (imputation) |
| Solution component created: | Data pipeline |

Table A2: Example of mapping TRIPOD reporting item 9 to a component and component task

Note that every guideline item was accounted for in the mapping. Where items were inapplicable to implementation process, such as administrative or research management elements, they were listed as such in the mapping (although excluded in the consolidated mapping table 5 provided in the main paper).

For step (3), an example of the consolidation is shown in Table A4.

| Raw mapping tasks | Final consolidated task |
| --- | --- |
| - Define & handle missing data (imputation) - Define and handle feature/predictor transformations - Perform other necessary data pre-processing steps | Transform Data |

Table A4: Example of consolidating multiple component tasks to a single component task

Based on the method above, each component comprises many tasks, each listed in Table D3 for each stage. In Figure 1 (main paper) we have summarized the state of each component as one of three: (i) design , (ii) development and test and (iii) updates. These three states reflect standard solution engineering steps, applicable to the development of any component. The timing of the state was determined by the need for the developed component, within the given stage. For example, the HCI was not required until the trial stage (IV), which meant design could occur in the previous stage and updates optionally could occur in following stages.

# Appendix B: Search Queries

## Pubmed Search Query

## EMBASE Search Query

## Web of Science Search Query


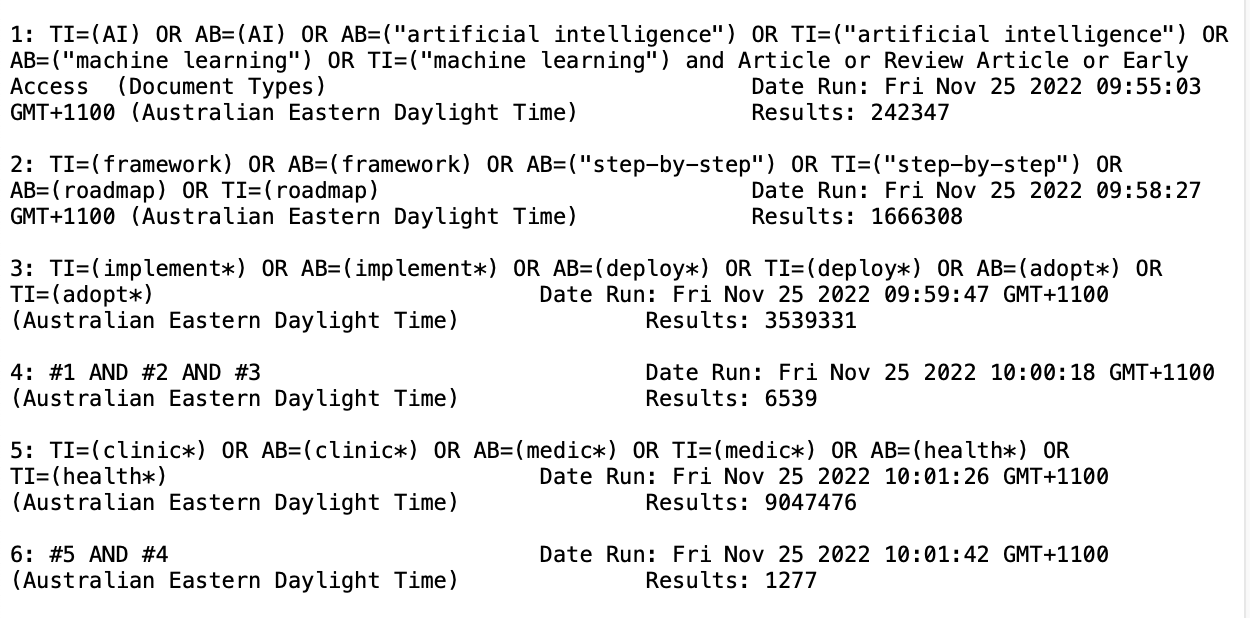


## CINAHL Search Query

Note this has been transcribed from the HTML of the print search history

## IEEExplore Search Query

# Appendix C: Eligibility Criteria

## Inclusion criteria:

1. hospital (acute) care setting

2. Broad AI framework applicable to many clinical AI tasks

3. Framework must include implementation of AI

4. Framework should impact clinical workflows

## Exclusion criteria:

1. Non-acute settings:

* primary care (GPs)

* Home care

2. Framework too specific

* Focused on one specific AI task

* Focused on a specialist/niche domain

3. Framework specific to imaging only AI

4. Framework does not include AI implementation component

5. Frameworks that do NOT impact clinical workflow, e.g., administrative only AI

6. Specific technology/IT 'frameworks', rather than generic

7. Not a framework: Where the authors do not propose a new framework

# Appendix D. Extracted data items

Paper information:

1. Title
2. Year
3. Authors
4. Country

Framework information:

1. Niche framework?: Is the framework generic to all healthcare or specific to a particular specialty or viewing implementation from a specific perspective
2. Clinical setting
3. Classification: process (stage-based), determinant (theme-based) or both
4. Classification detail: extra detail if the classification is not straight forward
5. Other relevant factors relating to the overall framework, for example is the paper directed at a particular audience
6. Basis framework: cited framework(s) the proposed framework is based on
7. Method detail: how the framework is derived
8. Method summary
9. Framework name
10. Number of themes
11. Number of sub-themes
12. Theme name used in the paper
13. General note on themes
14. Number of stages
15. Number of sub stages
16. Stage name used in the paper
17. Stage notes

Theme information captured by theme and sub-them:

1. Theme title
2. Sub-theme title
3. Description

Stage information captured by stage and sub-stage:

1. Stage title
2. Sub-stage title
3. Description

Note that during data capture, Gama et al.’s paper was an extension of Greenhalgh et al.’s NASSS framework, and therefore we included the NASSS framework within the data extraction to correctly cite the evidence found.

# Appendix E: Mapping Review Paper Stages/Themes to Provisional Framework

The themes and stages, including all sub-themes and sub-stages, that were captured in the data extraction from the scoping review were mapped to the stages and tasks of the provisional clinical AI implementation framework using an Excel spreadsheet. Where no or only partial mapping was possible, missing stage and/or theme elements were noted.

Theme mapping assigned coverage of full coverage (1), partial coverage (P) and no coverage (0) against each theme and sub-theme. Table E1 provides the full theme mapping.

In the stage mapping (provided in Table E2), the identified stages and sub-stages were firstly mapped to both the provisional SALIENT stages (I, II, III, IV and V) and SALIENT element E: Routine use, denoted ‘RU’ in the ‘Mapped Stage’ column. Where the identified stage was out of scope (for example, ‘making it to market’, a stage identified in Wiens et al.) the stage was noted as “OOS”. Secondly, the identified stages and sub-stages were also mapped to provisional SALIENT framework tasks and overall coverage listed. In some cases, it was implicit within the identified stage, that coverage was met without specific tasks being identified. For example, Bedoya et al.’s silent evaluation stage directly maps to SALIENT’s stage III. Implicit coverage was considered full coverage. Otherwise, the task coverage uses the same scoring approach as for themes.

## Table E1 (over 5 pages) : Review paper theme mapping to provisional framework tasks

Table E1: Author, theme and sub-theme mapped to provisional framework tasks listed (P1-P2, CW01-CW07, DP01 – DP10, AM01 – AM04, HC1-HC4, TS01-TS03, CS01-CS02, EM1-EM11). A coverage of: 1 means full coverage; P means partial coverage; and 0 means no coverage. Note that Greenhalgh et al themes from their NASSS framework were included as Gama et al. directly extended the NASSS framework and rather than list them under Gama et al. we directly attribute the themes.

## Table E2 (over 4 pages) : Review paper stage mapping to provisional framework tasks

Table E2: Author, stage number, stage and sub-stage mapped to provisional framework stage (Mapped stage column) and tasks listed (P1-P2, CW01-CW07, DP01 – DP10, AM01 – AM04, HC1-HC4, TS01-TS03, CS01-CS02, EM1-EM11). A coverage of: 1 means full coverage; P means partial coverage; and 0 means no coverage. Mapped stages are: I, II, III, IV, V, RU (routine use), XS (cross-stage), OOS (out of scope). Note that Greenhalgh et al stages from their NASSS framework were included as Gama et al. directly extended the NASSS framework and rather than list them under Gama et al. we directly attribute the stages in this mapping.

## Table E3: Summary of Mapping
